# Antiseptics

**Published:** 1896-08

**Authors:** Geo. E. Hunt

**Affiliations:** Indianapolis, Ind.


### THE DENTAL REGISTER.

VOL. L.] AUGUST, 1896. [No. 8.

### COMMUNICATIONS.

### Antiseptics.

BY DR. GEO. E. HUNT, INDIANAPOLIS, IND.

Read before the Indiana State Dental Society, Indianapolis, Ind., June 30, 1896.

In dentistry we find that antiseptics have grown steadily in their usefulness as our knowledge of their practicability has increased, until now scarcely a patient leaves our chair without having been subjected to the use of an antiseptic in one form or another.

There are several practical uses of these agents, the importance of which have been largely overlooked by many who are staunch supporters of antiseptics, and it is to these I desire to call your attention.

A mouth-wash is almost, if not quite, as important a component to one's toilet as any tooth powder, yet how seldom do we find such an article even in the possession of our most particular patients. And why is this?

It is either because the patients are ignorant of its value or because they willfully neglect their teeth. In any case the fault is largely our own, and may be ascribed to the fact that we either do not sufficiently urge upon our patients the importance of this valuable prophylactic measure, or because we underrate it ourselves.

The education of our patients is a subject in itself, and an important one, too, but we shall not encroach upon it except in so far as it treats of mouth-washes; of what they should consist, and when and how used, associated intimately, as it is, with the subject in hand.

You will observe that I have taken the liberty of assuming that we are all agreed upon the therapeutical merits of mouth-washes; hence we will not stop to consider the subject in this light.

In the first place, we should give each and every patient to understand that if he desires to preserve his teeth he should provide himself with a mouth-wash and use it regularly and liberally.

The ideal mouth-wash should possess these essential properties: antiseptic, alkaline, deodorant, somewhat astringent, yet entirely agreeable to the senses of taste and smell. Antiseptic, to arrest fermentation existing through presence of particles of food which remain lodged between and about the teeth; alkaline, in order to neutralize any acidity—the presence of which is so destructive to tooth-structure; deodorant, that any existing odor arising from fermentation, the use of tobacco, etc., may be destroyed or at least modified, thereby rendering the breath inoffensive. The astringent property of a mouth-wash should not exist in a marked degree, just sufficiently to assist in preserving a healthful condition of the gums and mucous membrane.

By combining a pleasant taste and fragrance to the preparation you endow it with properties which assist, perhaps, more than its real prophylactic worth in influencing patients to use it regularly.

Preparations known as antiseptic mouth-washes are numerous, and, like those for the relief of all the ailments to which flesh is heir, there are many which do not meet the requirements for which they are intended, while some have been proven to possess great practical therapeutical value.

Recommend to your patients a preparation, the merits of which you are thoroughly familiar with, or furnish them with a prescription composed of ingredients carefully selected to meet the requirements of the case.

The most essential time to use a mouth-wash is just before retiring. At this time the teeth should be properly cleansed by the use of the brush, the wash, properly diluted, furnishing the liquid with which the brush is saturated. After the use of the brush the mouth should be thoroughly rinsed with the wash. Patients should understand that there is no such thing as the abuse of a desirable wash, and that the mouth-wash habit, if such an expression is allowable, is a thoroughly desirable one, since cleanliness is next to godliness.

The importance of using preparations which are agreeable to the senses should not be lost sight of. All preparations used about the mouth, whether by injection or as a wash, gargle, or even for local application should possess these qualities. Our aim is to please, and pleasing signifies the creation of a favorable impression. We must save ourselve the embarrassment incident to unfavorable impressions arising from neglect of this small item.

The accomplishment of the desired end is within the grasp of all who will give it a little study. The addition of one of the balsamic agents to an otherwise offensive preparation will be accompanied with satisfaction to both patient and practitioner.

We should have on hand, for general use, an antiseptic which by different degrees of dilution will fulfill the requirements such as the term "general use" signifies, though we should in nowise look upon it as a "cure-all," since so many cases require special attention.

THE DISINFECTION OF INSTRUMENTS.

Many of us seem to feel that we have fulfilled all that the laws of hygiene and prophylaxis require when we see that our instruments are carefully wiped with a clean cloth or napkin, or perhaps dipped into water and then wiped dry. This may, and perhaps does, remove all visible stains and dirt, but we must remember that in all probability many germs remain upon their surfaces. Certainly the vast majority of these germs or micro-organisms are harmless, but on the other hand, those same instruments which have been replaced in your cabinet, supposedly clean and ready for use, may be infested with micro-organismes of the most infectious character. What an unpardonable crime it would be to affect a poor, unsuspecting patient with syphilis, for instance, through neglect of this sort when so convenient and sure a safeguard is at hand.

This safeguard should consist of a strong antiseptic solution, the active ingredients of which will not deteriorate by long standing. Bichloride of mercury corrodes steel, and therefore is not available for this purpose.

Here is another use for your general antiseptic previously referred to. Have at hand a vessel (a finger bowl is entirely suitable) containing your solution, and at the conclusion of each operation, irrespective of the social standing or general character of the preceding or succeeding patient, see that each instrument used is carefully immersed in this solution.

Closely allied to this prophylactic measure is the attention given our hands before introducing them into a patient's mouth. Considering that our hands are almost constantly within observation, it is apparent that it behooves us to be particular regarding their appearance. While it might not be especially observed that they are clean, it certainly would be noticed that they are *not*, if such be the case. In reality we have not done our duty entirely when we present ourselves with clean hands; they, too, should be disinfected.

Previous to beginning an operation, and preferably within view of the patient, the hands should be thoroughly washed with soap and water. A nail brush should be freely used in this connection. After all signs of stain and dirt have been removed, the hands should be carefully dried, and following this immersed in an antiseptic solution. To this add the cleaning of the nails and the use of a spray of toilet water from your atomizer, and you may go before your patient feeling that you have protected him (and yourself), against the danger of infection according to the most approved methods. It may be argued that the end does not justify the means, for to be sure the pursuance of this plan means an apparently unnecessary expenditure of time in many instances, but one needs only to draw a picture in one's imagination of a ruined and sacrificed life resulting from his neglect in disinfecting instruments or hands, to feel that the time consumed is fully compensated for by an amount of personal satisfaction derived from the knowledge that such a misfortune had not come to him on this account.

If we aspire to be "up to date" in our profession we must avail ourselves of "up to date" methods, and this is one of them. The surgeon of to-day spares no pains in fullfilling every detail which comes within the category of antiseptic precaution. So we, as dental surgeons, must practice in the same channel if we are specialists in medicine.

The future of antiseptics can hardly be over-estimated since we have come into a full knowledge of their true value. Their prophylactic worth is not sufficiently understood by the public generally, and it remains for us to distribute such knowledge upon all sides. By so doing, and not until then, do we fulfill our duty to humanity.

